# How Do Academic Elites March Through Departments? A Comparison of the Most Eminent Economists and Sociologists’ Career Trajectories

**DOI:** 10.1007/s11024-020-09399-1

**Published:** 2020-03-13

**Authors:** Philipp Korom

**Affiliations:** Department of Sociology, University of Graz, Universitätsstrasse 15/G3, 8010 Graz, Austria

**Keywords:** Academic elites, Academic careers, Nobel Laureates, Sequence analysis, Elite networks

## Abstract

This article compares the career trajectories and mobility patterns of Nobel Laureates in economics with those of highly cited sociologists to evaluate a theory advanced by Richard Whitley that postulates a nexus between the overall intellectual structure of a discipline and the composition of its elite. The theory predicts that the most eminent scholars in internally fragmented disciplines such as sociology will vary in their departmental affiliations and academic career paths, while disciplines such as economics with strong linkages between specialties and shared standards of excellence will be dominated by a more homogeneous elite. The comparison provides strong empirical evidence in favor of Whitley’s theory. The careers of the most eminent economists are closely tied to the top five departments of the discipline, whereas the career pathways to eminence in sociology are largely unpredictable.

**Electronic supplementary material:**

The online version of this article (10.1007/s11024-020-09399-1) contains supplementary material, which is available to authorized users.

## Introduction

This article sets out to test selected propositions of a ‘theory of middle range’ (Merton [1949] 2007) put forward by Richard Whitley in his article “Umbrella and Polytheistic Scientific Disciplines and their Elites” (Whitley 1976). Whitley based his theory on two basic postulates: First, the ways in which specialties relate to disciplines produce different cognitive structures of individual disciplines. Second, there is a specific nexus between the cognitive structure of a discipline and the configuration of its elite. While these postulates are interrelated, I will only subject the second one to empirical scrutiny.^1^

The concept of a scientific discipline is an ambiguous one; the concept of a specialty is not. What differentiates specialties is the object of study. For example, there are many ‘sociologies of’ something – social problems (e.g., poverty and inequality), social institutions (e.g., family), or social groups (e.g., race and ethnic minorities). Specialties can cluster into disciplines, which are not problem-defined but rather perspective-defined. That is, a discipline “is organized around and essentially differentiated from other disciplines by one or more predominant perspectives from which a number of different problems are viewed” (Akers 1992: 4). Economists and sociologists, for example, have equally studied capitalism, albeit through different lenses. Sociologists have been interested in capitalism mainly for its social effects while economists have been drawn to the quest of discovering its key driving forces (Swedberg 2005). More generally, one could argue that “to be a member of a discipline is to adhere to a particular approach to scientific understanding applied to an aspect of ‘reality’” (Whitley 1976: 473). Disciplines have two major components: An intellectual component, or the forms and kinds of knowledge the discipline wants to enhance, and an occupational component, where disciplines are institutionalized in research institutions and its members are bound to one another by a professional association (Zald 1991: 168).

The first part of Whitley’s theory makes a distinction between two types of disciplines. Whitley uses the label ‘polytheistic’ to refer to disciplines with low consensus on what constitutes good science, in which “specialties […] are unlikely to be institutionalized as foci of commitment and identity” (p. 478). In contrast, the label ‘umbrella disciplines’ describes disciplines with shared beliefs on how to build knowledge, in which the “research production is predominantly organized at the specialty and research area levels without direct reference to, or influence from, the discipline.” Umbrella disciplines merely act as “loose ‘holding’ organizations for diverse specialties” (p. 476).

The second part of Whitley’s bipartite theory offers propositions about elite formation. As divergent views become institutionalized in university departments, “organizational authority and intellectual authority overlap [….] considerably” in ‘polytheistic disciplines’ (p. 485), which leads to heterogeneity among elites. In ‘umbrella disciplines,’ however, where specialties and *not* individual departments are key to the cognitive organization, elites are expected to concentrate within a few core specialties of a discipline. Thus, we see high levels of elite homogeneity.

While many scholars have adopted Whitley’s typology of scientific disciplines,^2^ his core claims on elite formations are rarely cited and have never been tested. What is more, the pertinent literature on scientific elites (Graf 2015; Sternberg et al. 2016) does not inform us about interdisciplinary differences. Theoretical knowledge alone, however, does not suffice for the accumulation of reliable knowledge. To assess the validity of Whitley’s theory, I will proceed in three steps. First, I will aim at a closer understanding of the central propositions in Whitley’s theory. Second, I will discuss sociology and economics as representatives of the polytheistic and umbrella types of social sciences disciplines. Third, I will analyze the careers of the most eminent economists and sociologists and ask if the empirical insights gained are consistent with Whitley’s theory. The article concludes with a discussion of elites and their influence in different social science disciplines.

## Dissecting Whitley’s Theory

Whitley’s essay (1976) is written in a very abstract style (with phrases such as ‘ordering principle,’ ‘explanatory models,’ ‘units of organizations,’ ‘disciplinary definition’). Thus, his concepts first need to be unpacked and clarified.

The role of specialties within a discipline is the central variable Whitley uses to distinguish between disciplines. There are two extremes: Specialties are promoted to such a degree that they end up being quite autonomous from the master discipline, or research in specialties remains within the discipline’s boundaries and pursues questions related to its overall approach. This difference stems from the relative structuring power of what Whitley calls the discipline’s ‘ordering principle,’ i.e., (epistemological) beliefs about how to conceive problems and order them into a general framework.^3^ From these considerations, the first proposition can be derived:

### **Proposition 1**

*The more developed and widely accepted the discipline*’*s ‘ordering principle’ is, the more likely the discipline is to have specialties that function autonomously.*

In ‘polytheistic disciplines,’ scholars frequently debate the very definition of the discipline (establishing its boundaries, legitimate methodologies, and so forth). The dominance of *multiple* beliefs on the best path to ‘truth’ leads to a close interplay between the discipline and its specialties, which do not become highly institutionalized. In contrast, there is consensus within ‘umbrella disciplines’ about what constitutes good science. The ‘structuring principle’ is merely refined in the various specialties, which become more easily institutionalized and develop mostly autonomous from their home discipline.^4^

Whitley further argues that specialties within disciplines, the department in which specialties are located, and specialist factions within departments, are locked in a continual struggle for authority, status, and power. In ‘polytheistic disciplines,’ a variety of departments carry out the crucial authority roles of training neophytes in the discipline and subsequently hiring or promoting them, thus acting as “the dominant unit of research organization and control” (p. 488). In ‘umbrella disciplines’ that are unified under one cognitive goal and have highly institutionalized specialties, the authority lies more with specialties than with departments. Hence:

### **Proposition 2**

*The existence of highly institutionalized specialties (in ‘umbrella disciplines’) weakens the authority positions of departments.*

Another central postulate is that ‘umbrella disciplines’ have a prestige hierarchy between specialties, as some specialties are viewed to be more fundamental to the discipline than others. Core specialties are, inter alia, predicted to also obtain more research facilities than marginal specialties.

### **Proposition 3**

*Disciplines with highly institutionalized specialties (‘umbrella disciplines’) are hierarchically structured. Core specialties dominate marginal specialties.*

This hierarchical order of ‘umbrella disciplines’ allows us to locate elites. Elites work in ‘core specialties’ and act as the ‘guardians and interpreters’ of the ‘ordering principle’ (p. 488). Elites in these core specialties tend to have discipline-wide influence, with their categories of thinking considered to be models for other scholars to emulate. In ‘polytheistic disciplines,’ in contrast, where the internal hierarchy is only loosely institutionalized, elites may work in very different areas of knowledge production. Their influence is rather limited and mostly bound to their home department.^5^

### **Proposition 4**

*In disciplines with highly institutionalized specialties (‘umbrella disciplines’) elites work in core specialties, while in disciplines with less institutionalized specialties (‘polytheistic disciplines’) elites specialize in very different fields of study.*

Departments play a crucial role in Whitley’s theory. In a section on ‘elites in umbrella disciplines,’ he states that “given the inequitable allocation of resources across universities, we would expect work in central specialties to occur mostly in the more prestigious departments, and these departments to concentrate on problems in basic specialties” (p. 489). Given proposition 4, it follows that elites in ‘umbrella disciplines’ concentrate in the top departments of the discipline. No such statement is made regarding ‘polytheistic disciplines.’

### **Proposition 5**

*In ‘umbrella disciplines’ elites work in the top departments of the discipline, while in ‘polytheistic disciplines’ elites may be affiliated with departments of very different status.*

Whitley further argues that the internal structure of disciplines impacts the mobility of elites: “Scientists working in a major field can easily move to a department where resources are concentrated.” He adds: “In contrast to polytheistic disciplines, personal mobility between organizations is easier […]” (p. 489). Translated into concrete terms, given the fact that top departments in ‘umbrella disciplines’ specialize in core specialties that share common analytical methods, elite members can easily switch positions between top departments. Elites in ‘polytheistic disciplines,’ in contrast, are more likely to face hiring obstacles, as there is little agreement on what counts as academic excellence (or lack thereof). As hiring processes become comparatively random, elites are more likely to circulate between departments of different prestige.

### **Proposition 6**

*In ‘umbrella disciplines’ elites circulate primarily between top departments, while in ‘polytheistic disciplines’ elites circulate between departments of different prestige.*

Taken together, these six propositions form a theory on elite formation that can be tested. I will confine this article to a test of predictions that can be derived from propositions 5 and 6.

## Sociology and Economics as ‘Polytheistic’ and ‘Umbrella’ Disciplines

In Whitley’s article (1976), the actual sciences get only little or cryptic mention. However, Whitley does think of sociology as a good exemplar of a ‘polytheistic discipline’ and of physics as embodying the key characteristics of an ‘umbrella discipline.’ As I aim to study the social sciences, I will focus on sociology and economics, the latter arguably being the social science discipline that comes the closest to meeting the key criteria of an ‘umbrella discipline.’

### Sociology – A Discipline Without Core

While it is true that sociology between the 1940s and 1960s was marked by a few paradigms (Turner 2014), it is often argued that functionalism and systems theory lost their near-exclusive hold in the early 1970s. Even if there are criticisms of such a narrative as a ‘folk theory’ of American sociology’s postwar development (Calhoun and VanAntwerpen 2007), it is generally accepted that baby-boomer scholars pouring into the discipline in the 1960s and 1970s owed little allegiance to the established fields in sociology (Wallerstein 2007), and that a discipline-wide consensus on how to achieve progress vanished. Today, “sociology appears to be one of the most internally divided disciplines, if not the most” (Lipset 1994: 199). Covering an enormous topical terrain, sociology as a discipline is best conceptualized as a ‘polymorphic population’ (DiMaggio 1997: 189). Because of a lack of intellectual and organizational direction from the discipline, leading sociologists tend to split into different ‘camps’ with very different institutional bases, which is empirically demonstrated in two studies. Phaedra Daipha investigated joint memberships in sections of the *American Sociological Association* and analyzed section clusters. The analysis reveals a picture of sociology that is a “considerably diffuse scientific community with research networks radiating around conceptually relevant intellectual and organizations centers” (Daipha 2001: 86). Based on co-citation data generated from the *Social Science Citation Index* (SSCI), Diana Crane and Henry Small visualized cross-linkages between specialties in sociology and economics and found the intellectual structure of sociology to be much more diffuse. In contrast to economics, sociology lacked a “sizeable core that incorporated a number of major subfields” (Crane and Small 1992: 222). Because of the fractured cognitive structure sociology is not “able to argue with one voice about what is *elementary*. This will be true even if some leading sociologists will be recognized as clearly elite by many (never all) people from other disciplines, as for example Pierre Bourdieu or James S. Coleman would be” (Stinchcombe 1994: 80).

### Economics – A Discipline Divided into Specialties and Dominated by Core Theories and Methods

While it is commonly argued in sociology that every existing theory has a morsel of truth (Sztompka 2010), the ‘neoclassical paradigm’ has become *the* dominant paradigm in economics from the 1960s onwards. In addition, the mathematization of economics since the end of the Second World War has created a bond that ties economists together. Mathematical models have become widely accepted as the ‘gold standard’ of the discipline (Debreu 1991).

Applying bibliographic coupling to 415,000 documents published between the 1950s and 2014, Claveau and Gingras (2016) trace the macrodynamics of economics. In their approach, the authors assume that similarities among references in different texts are an appropriate indicator of proximity and thus of belonging to a given specialty. The study brings to the forefront recognizable specialties that endure for longer or shorter periods. Most importantly, the network is always marked by core specialties. In the mid-1980s, for example, the two specialties having by far the most intense connections to each other and to the rest of the network are the ‘econometrics-centered specialty’ and the ‘macro/money specialty’ (Claveau and Gingras 2016: 580).

Moreover, economics is marked by an extremely hierarchical structure. Marion Fourcade, for example, argues that “the top American economics departments represent the vast majority of the authoritative work produced by the discipline, and they exercise a considerable amount of hierarchical control over the rest of the field” (Fourcade 2006: 171). In no other discipline can one find the extraordinary volume of data and research on rankings (of journals, departments, and individuals) produced by economists (Fourcade et al. 2015).

Given this existing evidence, comparing sociology to economics to test some components of Whitley’s theory appears highly appropriate. Sociology is a fractious discipline with ambiguous and heterogeneous standards of evaluation. Economics, in contrast, is marked by a more cohesive and hierarchical organization.

## Hypotheses

To test the second part of Whitley’s bipartite theory, I will not only investigate the main institutional affiliations of elites in sociology and economics but also develop a holistic perspective of the elites’ ‘institutional’ and ‘external’ careers. Both types of careers are conceptualized as “successions of related jobs arranged in a hierarchy of prestige through which persons move in an orderly sequence” (Wilensky 1961: 523). The ‘institutional’ strand encompasses, for example, professorships in universities or research institutions (e.g., Max Planck Institutes in Germany) where elites work full-time, while the ‘external’ strand refers to short-term fellowships and visiting professorships outside of the professor’s chief institution (see chapter 2 in Light, Marsden and Corl (1973)).

*Entrance into academic careers*: Given the existing level of social exclusiveness in all the social sciences, the prestige of PhD-granting institutions is generally found to be decisive for the prospects of academic professionals (Burris 2004). Given the more cohesive structure of economics that is dominated by elite institutions, however, I expect to find more eminent economists than sociologists with PhDs from elite departments (Hypothesis 1a).

Further, it is well established that the discipline usually corresponds to the entry-level basic (BA) training in a university department, while the specialty corresponds more to the research level earned at PhD levels (Hagstrom 1965). Given that specialties are only highly institutionalized in economics, I expect elite economists to transition after the PhD more quickly to the first (specialty) professorship than sociologists (Hypothesis 1b).

*Career sequences:* In the case of institutional careers, the normal succession of positions after receiving a doctorate is assistant professor, associate professor, and professor. The focus here is on professorships only.^6^ My intent is to explore which departments first hire scholars who later become eminent scholars, and which departments attract these scholars once they have gained tenure. The focus is on career trajectories, which implies that appointments are not treated as isolated from each other. I expect to find that eminent economists march predominantly through elite departments, while eminent sociologists are affiliated with a more diverse group of departments throughout their working life (Hypothesis 2).

*Upward and downward mobility*: While in general we expect elite careers to continue advancing on a path toward eminence, we must bear in mind that elite scholars can easily take their talents elsewhere as tenured professors. It is thus easy to imagine—and it can indeed be observed—that an eminent sociologist like Manuel Castells can move from an elite department (UC-Berkeley) to a less prestigious one (University of Southern California) to reduce teaching obligations, for example. Observations like this suggest that both upward and downward mobility across lifetime careers should be considered. Based on Whitley’s conceptual model, I conjecture that eminent sociologists will move significantly more between elite and non-elite departments. Hence, multidirectional mobility in elite strata is more characteristic for sociology than for economics (Hypothesis 3).

*Transition between professorships*: Besides the order of career sequences, temporality appears a crucial aspect of occupational trajectories. As Whitley’s theory implies, economics professors have more flexibility to move between departments than sociology professors because of their shared epistemological/academic standards. Empirically, I expect to find that there are fewer years between professorial appointments at different departments in economics than in sociology (Hypothesis 4).

*Fellowships/visiting professorships*: From time to time, elite scholars leave their home institution to pursue studies elsewhere. The ‘visiting scholar or fellow’ has become, for example, a familiar figure at major universities (Mayntz 1960). It is reasonable to deduce the same predictions from Whitley’s theory for the institutional *and* the external careers of academic elites. I thus expect economists to circulate more frequently via visiting scholarships and professorships between elite departments than sociologists (Hypothesis 5).

## Elite Identification, Data Sources, and Data Preparation

### Elite Identification

A common strategy to identify elites is the ‘reputational method’ (Hoffman-Lange 2018). First, leading experts of different knowledge domains are asked to help compile a comprehensive roster of scholars who have significantly advanced scientific knowledge. Then an expert committee (often aided by advisers) votes for one, or several, winners. In general, that is how the ‘The Sveriges Riksbank Prize in Economic Science in Memory of Alfred Nobel,’ the *ne plus ultra* prize of the discipline, was awarded — 49 times to 79 Laureates between 1969 and 2017 (for the foundation’s history, see Offer and Söderberg 2016).^7^ All 79 Laureates will be considered in this study.

Identifying the elite for social science disciplines such as sociology is a far greater challenge. Eminent sociologists are honored with various awards such as the ‘Holberg Prize.’ However, even if there is considerable prize money at stake, these awards are not intended as surrogates for the Nobel Prize and have not changed the discipline’s reward system in any fundamental way. Thus, one must settle on an alternative ‘Geiger counter’ of recognized outstanding achievement: citation counts. The principal justification for this choice is that while publications might be cited for reasons other than quality, this is unlikely to be the case for the most cited scholars (Parker et al. 2010).^8^

The methodology adopted here does not assume that mere citation counts allow one to establish a strict, linearly ordered system of excellence. Furthermore, it departs from conventional citation analysis by disaggregating citation scores at the level of academic journals. Disaggregation allows us to investigate, besides the number of citing articles, a scholar’s citation impact across different nations and specialties. The ‘prestige elite’ (Korom 2020) is not solely equated with the most cited scholars of the discipline but rather with those thinkers whose ideas have permeated the most national sociologies and specialties within the balkanized discipline of ‘sociology.’^9^

To identify elites, I have consulted the *Social Science Citation Index* (SSCI, 1956-) included in the *Web of Science Core Collection* and conducted three steps:^10^ (1) Considering all available previous rosters of eminent sociologists (i.e., Cronin et al. 1997), entries on key thinkers in major encyclopaedias (i.e., Wright 2015) and *Google Scholar* citation statistics, I compiled a list of 194 individuals with more than 1,000 citing articles in SSCI-indexed sociology journals (see Appendix A1 in the Online Supplementary Material). (2) 36 sociology journals were selected, of which two stand for either one of the eight national sociologies or one of the ten specialities considered (see Appendices A2 and A3 in the Online Supplementary Material). For each national sociology and speciality, I calculated who belongs to the uppermost quintile (‘top 20%’). (3) A three-dimensional cube was constructed with the x-dimension indicating the number of top quintiles in national sociologies, the y-axis indicating the number of top quintiles in specialties, and the z-axis indicating the total number of citing articles in the 36 sociology journals considered. Projected onto this cube are only scholars who belong to the top 20% of the citation distribution of three national sociologies *and/or* three specialties *and* who were born after 1850.^11^

**Fig. 1:**
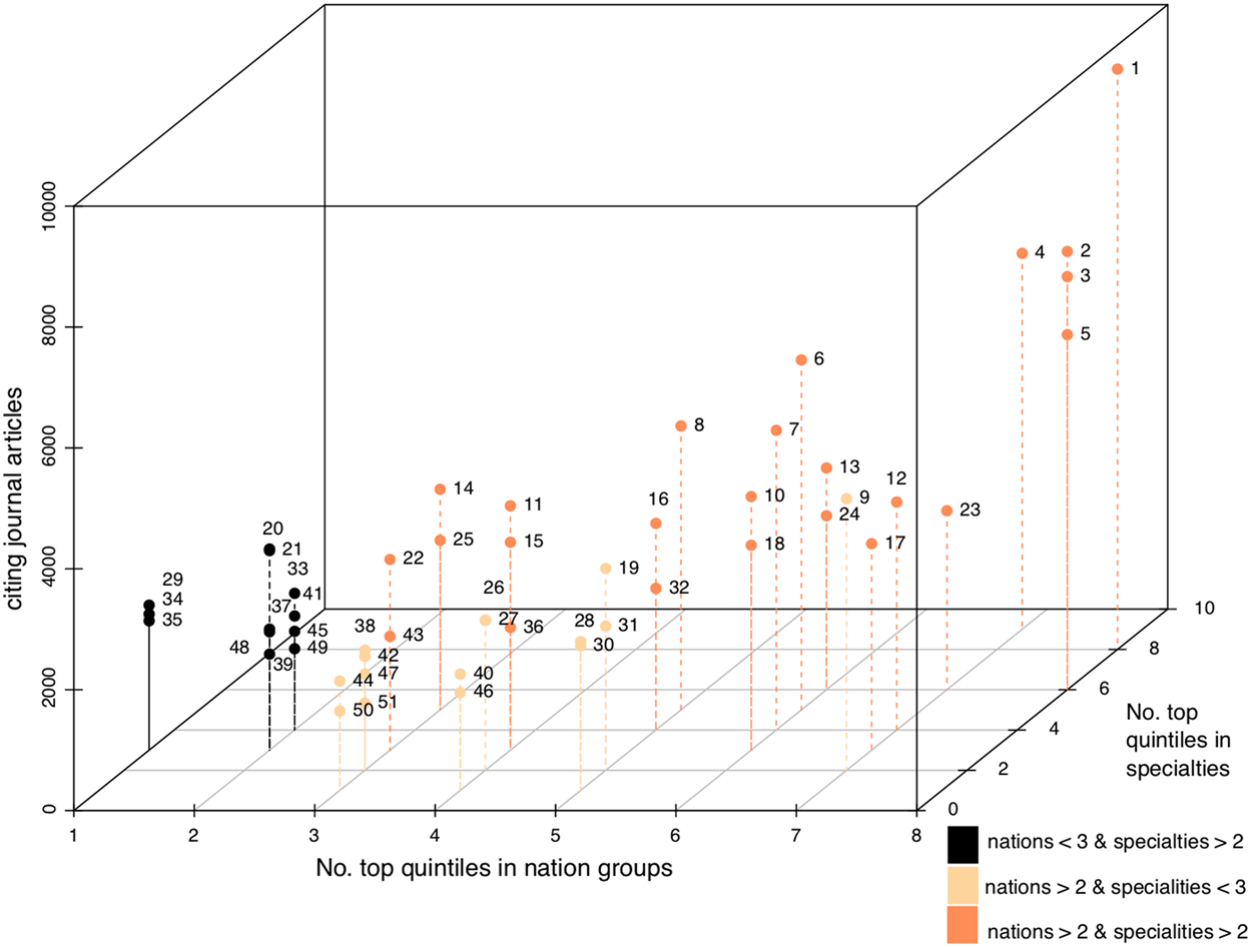
A three-dimensional depiction of eminence in sociology. **Notes**. Each number stands for a scholar (see Appendix A4 in the Online Supplementary Material). 1 stands for Pierre Bourdieu, 2 for Erving Goffman, 3 for Talcott Parsons, 4, for Anthony Giddens, 5 for Robert K. Merton, etc. Pierre Bourdieu is located at the upper right corner because of his extraordinary overall citation count (9,601 citing articles) and the fact that he belongs to the top twenty most cited scholars in eight (out of eight) nation groups and eight (out of ten) specialties

Figure 1 depicts the 51 scholars who fulfill both selection criteria. 24 belong to the top 20 most cited scholars in at least three nations *and* at least three specialties (right side of the cube); 15 belong to the top 20 most cited scholars in at least three nations (center of the cube); 12 are part of the top 20 most cited scholars in at least three specialties (left side of the cube). Most, if not all, of these 51 scholars are commonly considered to be ‘key sociological thinkers’ (Stones 2017).

### Data Sources

This study is based on a comprehensive biographical database created by the author. Biographical data were gathered from diverse sources. In the case of economists, official biographies can be found on www.nobelprize.org. However, these biographies rarely contain detailed information on the scholars’ time spent in different employment. I have thus relied first and foremost on the curricula vitae of economists and sociologists where these are available from personal webpages.^12^ Various editions of the “Who’s who in economics” have proved invaluable as well (Blaug 1999). I also consulted various biographical dictionaries (Vane and Mulhearn 2005; Breit and Hirsch 2009).

For sociologists, I have relied on curricula vitae, American National Biographies, biographical entries in the Encyclopedia Britannica, and the International Encyclopedia of the Social and Behavioral Sciences, as well as on historical and more current editions of *Marquis* Who’s Who in America. Other important sources were ‘biographical notes’ contained in the University of Chicago Library guides to the archival papers of a certain eminent sociologist, online biographies on the authors of 50 classics in sociology (available in the Austrian Archive for the History of Sociology),^13^ ‘biographical memoirs’ of scholars from the National Academy of Sciences, profiles of ASA presidents and obituaries in the ASA outlet footnotes, as well as biographies and autobiographies.

### Data Preparation

I have analyzed academic careers using sequence analysis (SA), which enables me to detect and classify overarching sequence patterns based on the timing and order in which elements appear (Gauthier et al. 2014). The SA data files use the so-called vertical ‘time-stamped-event’ (TSE) representation that list the ‘events’ experienced by each individual together with the time at which the events occurred. Sequences of events can easily be constructed from this representation. As shown in Table 1, the ‘events’ of interest are professorships and the ‘time’ is the age at appointment. The name of the department and its prestige rank are also registered (see Appendix A5 in the Online Supplementary Material). Prestige ranks for economics department are drawn from Amir and Knauff (2008), and ranks for sociology departments are from the 2011 QS World University Rankings. Amir and Knauff’s equate the prestige of a department with the sum of the ‘values’ of its PhD graduates, as reflected in the ‘values’ of their current employing departments. As the same methodology has never been applied to sociology departments worldwide, I have used the ranking of the company *QS Quacquarelli Symonds* that is based on a mix of measures (e.g., citations per faculty, survey information).^14^ Despite some evidence that departmental prestige tends to stay rather stable over time (Weakliem et al. 2012), we would ideally draw on longitudinal prestige data. As such data is not available, however, I must use cross-sectional data, while treating them with much caution. Therefore, I have based the analysis on prestige groups, thereby treating department ranks simply as orders of magnitude.

**Table 1:** Snapshot of the sequence data set (TSE-format)

timestamp	event/1	event/2	age	time	ID	name	discipline
1978	rank 11–20	LSE	38	38	1	Akerlof	Eco
1980	rank 6–10	UC-Berkeley	40	40	1	Akerlof	Eco
2014	rank 21 or lower	U of Georgetown	74	74	1	Akerlof	Eco
…	…	…	…	…	…	…	…
1981	rank 6–10	UC-Los Angeles	34	34	81	Alexander	Soc
2001	rank 6–10	Yale U	54	54	81	Alexander	Soc

Further, scholars might leave their home institution temporarily and spend time as a ‘visiting fellow,*’* ‘visiting professor,’ etc., at another research institution such as a university, an Institute of Advanced Study (e.g., the IAS in Vienna), or a foundation dedicated to research (e.g., Russell Sage Foundation). I decided to consider only those stays that lasted at least one full academic term (4–5 months).^15^ Registering all documented stays for each scholar allows me to construct a data set with the format outlined in Table 2.

**Table 2:** Snapshot of the social network data set

home institution	host institution	year	ID	name	discipline
UC-Berkeley	IAS	1967	1	Akerlof	Eco
UC-Berkeley	Indian Statistical Institute	1983	1	Akerlof	Eco
…	…	…	…	…	…
UC-Los Angeles	IAS	1985	81	Alexander	Soc
UC-Los Angeles	Swedish Collegium	1992	81	Alexander	Soc
UC-Los Angeles	University of Bordeaux	1994	81	Alexander	Soc
UC-Los Angeles	IHS, Vienna	1995	81	Alexander	Soc
UC-Los Angeles	CASBS	1998	81	Alexander	Soc
Yale U	U of London	2007	81	Alexander	Soc
Yale U	U of Cambridge	2012	81	Alexander	Soc

## Results

### Socio-demographic Profiles, PhD-Granting Institutions, and Transitions to First Professorship

Table 3 provides a general description of the eminent scholars’ profiles in the two disciplines. At first sight we see minimal differences between both populations. The age structure is quite similar across disciplines, and there is only one woman in each elite group. To investigate national backgrounds, we can differentiate between country of birth and country of residence, the latter referring to the country in which the scholar spent most of his academic life.^16^ In both disciplines, more than half of the scholars were born in the United States, and most of the remaining economists and sociologists were born into European families. Results for countries of residence underline the dominating role of the United States.

**Table 3:** Elite profiles in two disciplines

	economics		sociology
**year of birth (average**)	1928		1933
**male**	78		50
**country of birth**			
AU-CY-DE-FR-I-NL	11	AU-DE-FR-ES	12
British West Indies	1	Cuba	1
Canada	3	Denmark-Norway-Sweden	3
Finland-Norway-Sweden	6	Poland	1
Hungary-USSR-Russia	3	United Kingdom	3
India	1	USA	31
Israel	1		
United Kingdom	8		
USA	45		
**country of residence**			
FR-DE-NL	5	France-Germany-Spain	8
Israel	1	Sweden	1
Norway/Sweden	4	United Kingdom	4
USSR-Russia	1	USA	38
United Kingdom	5		
United Kingdom/USA	1		
USA	62		
received PhD from …*			
Carnegie Mellon University	4	Columbia University	7
Columbia University	4	ENS	2
Harvard University	9	Harvard University	5
M.I.T.	11	UC-Berkeley	4
Princeton University	4	University of Cambridge	2
Stanford University	2	University of Chicago	9
University of Cambridge	3	U of Wisconsin-Madison	4
University of Chicago	8	University of Paris	2
*other universities*	*30*	*other universities*	*16*
professorships per scholar**			
1 professorship	23	1 professorship	20
2 professorships	26	2 professorships	17
3 professorships	18	3 professorships	9
4 professorships	9	4 professorships	5
5 professorships	1	5 professorships	0
6 professorships	2	6 professorships	0
**years between PhD and 1**^**st**^**prof**.			
median	6.0	median	11.0
mean	6.66	mean	10.35
**total**	**79**		**51**

*The following economists and sociologists never received a PhD: J. Hicks, L. Hurwicz, J. Meade, and R. Stone

**John Nash never held a professorship

When we look at the PhD institutions of the Nobel Laureates in economics, M.I.T., Harvard, and Chicago have clear leading positions. A more fine-grained analysis^17^ reveals that M.I.T. is the most important ‘breeding ground’ for younger economists. A significant number of older eminent sociologists received their PhD from Chicago, Columbia, and Harvard. The more recent superstars in sociology such as A. Giddens or P. Bourdieu were partly trained in European elite institutions (Cambridge University, École Normale Supérieure) or at the University of California, Berkeley. Generally, one can conclude that elite institutions play a similar role in both disciplines regarding PhD training.

If we consider the number of professorships, similarities prevail over differences once again. In both disciplines, most scholar either stay at the department where they were promoted to full professorship or change professorship only once. Fewer scholars switch departments three or more times. In economics, however, there are three Nobel Laureates who continued their academic wanderlust even after the fourth professorship.

Another important finding gained from Table 3 is that the bulk of all economists have transitioned to their first full professorship about seven years after achieving their PhD, while half of all sociologists have not been promoted to the rank of professor, even 11 years after their PhD.

### The March Through Departments

To depict the diversity of career trajectories and track each individual pattern, I use a ‘decorated parallel coordinate plot,’ as introduced by Bürgin and Ritschard (2014). In such a visual display, each line represents a unique ordered pattern and the line width reflects the frequency of the pattern. The lines are jittered to avoid overlapping and to help identify typical patterns; patterns with frequency below the minimum support of 3% are grayed out. To facilitate the tracking of distinct patterns, there are gray arrangement zones at the intersection of the x coordinate (events) and the y coordinate (departmental rank groupings). Events in an ordered pattern are represented by solid squares.

**Fig. 2:**
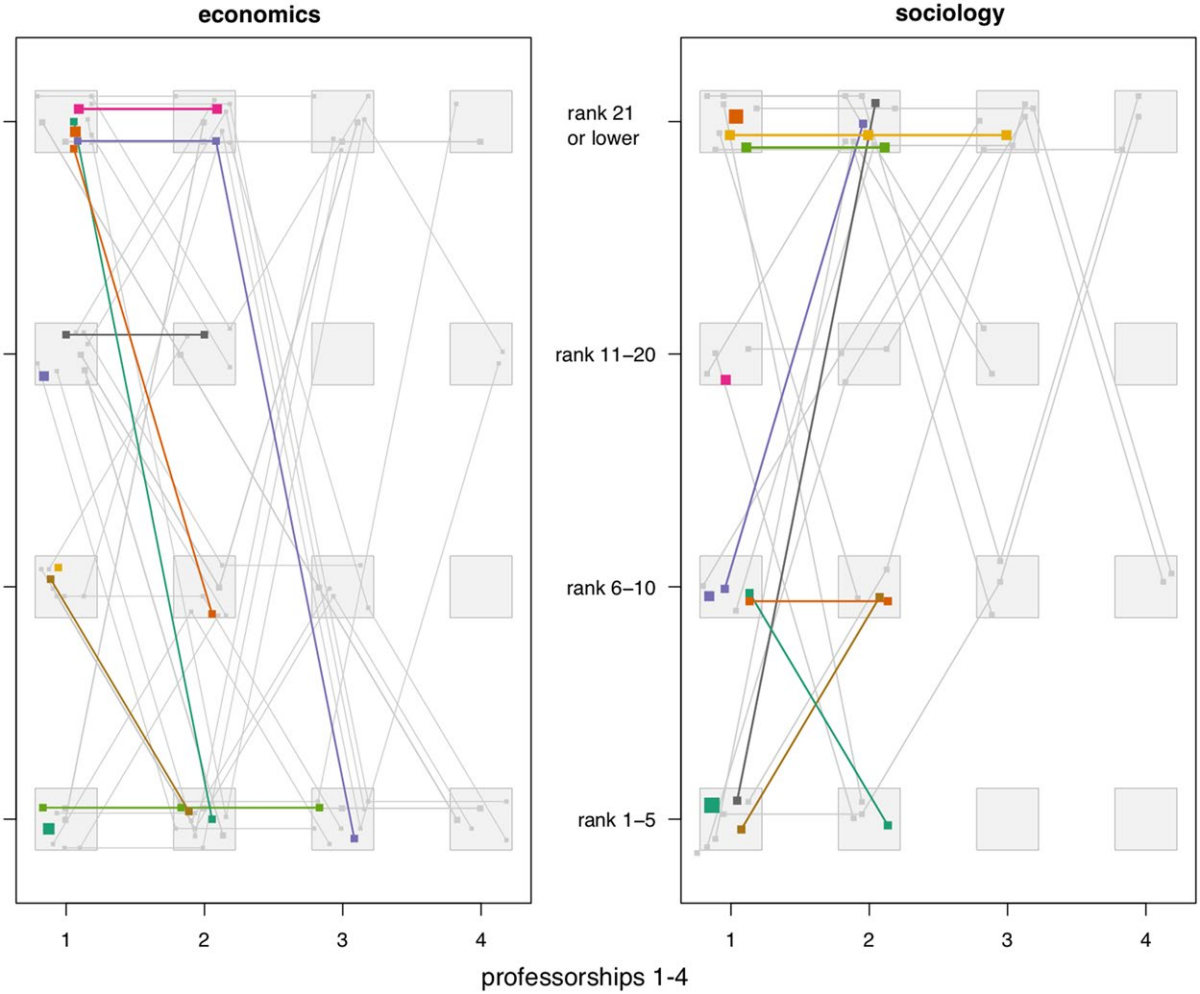
Career trajectories clustered by departmental prestige ranks. **Notes.** For economics, the rankings are taken from Amir and Knauff (2008), and for sociology, from the QS World University Rankings by Subject 2016 edited by *Quacquarelli Symonds* (see also Appendix A5 in the Online Supplementary Material)

Looking at the colored lines in Fig. 2, we learn that four out of the seven most frequent patterns lead to the top five economics departments (Chicago, M.I.T., Harvard, Stanford, Yale). We can, for example, derive that a commonly experienced pattern is to hold two consecutive professorships at departments with ranks below 20 and then be appointed professor at a department with the highest prestige (ranks 1–5).

The two plots in Fig. 2 are significantly different. For sociology, we find six frequent career trajectories, two of which are limited to less prestigious universities with rank 21 or lower. Three dominant trajectories are associated with declining prestige ranks, and only one stands for upwards mobility into the most prestigious departments (ranks 1–5). In addition, many eminent sociologists tend to stay in departments that belong to the same prestige group.

The exploratory plot suggests that norms in the organization of career trajectories differ across disciplines. Economists follow more standardized career paths that lead to the very top, while horizontal rather than vertical career growth seems typical for sociologists.

### Upward and Downward Mobility Across the Entire Career

To shed more light on mobility patterns, I probe whether the degree of upward and downward mobility varies across different stages of life. Table 4 considers all professorial moves between departments of different prestige rank for two age groups. Upward or downward mobility is coded if departments differ by more than five prestige ranks.

**Table 4:** Career mobility moves of professors by discipline

	economics			sociology		
	all	below age of 50	50 or older	all	below age of 50	50 or older
move up	31	25	6	14	8	6
move down	21	8	13	17	6	11
move within 5 ranks	51	35	16	19	12	7
*moves up*						
into ranks 1–5	16	12	4	3	3	0
into ranks 6–10	6	4	2	6	4	2
into ranks 11–20	6	6	0	2	0	2
into ranks 21–30	2	2	0	1	0	1
into ranks 31–40	1	1	0	0	0	0
into ranks 41–50	0	0	0	2	1	1
*moves down*						
into ranks 6–10	1	0	1	1	0	1
into ranks 11–20	4	2	2	1	1	0
into ranks 21–30	7	2	5	4	2	2
into ranks 31–40	0	0	0	2	1	1
into ranks 41–50	1	1	0	1	0	1
into ranks >50	8	3	5	8	2	6

**Notes.** For economics, ranks are taken from Amir and Knauff (2008), and for sociology, from the QS World University Rankings by Subject 2016 edited by *Quacquarelli Symonds* (see also Appendix A5 in the Online Supplementary Material)

It becomes apparent that in the case of economics, career moves are strongly associated with upward mobility; most scholars change affiliation to take up another professorship at one of the top five economics departments. However, in the case of economics professors above the age of 50, downward mobility becomes more frequent. To understand these results, it is important to recognize that American law granted the first exemption for postsecondary institutions to enforce mandatory retirement (at age 70). Following a review in the early 1990s, Congress then allowed the exemption to expire, and mandatory retirement was finally eliminated completely in 1994. Many Nobel Laureates who remained employed into their 70s and even 80s switched to less prestigious faculties. The best illustrative case is the Harvard-based economist W. Leontief, who joined New York University at the age of 70.

In sociology one finds only partly similar mobility patterns. As in economics, downward mobility predominates towards the end of sociologists’ occupational careers. There are, however, also striking differences between both disciplines. In sociology, there is a near balance between upward and downward career moves before the age of 50. Moreover, the share of scholars entering elite departments (ranks 1–5) is much lower in sociology than in economics, even if one considers only the first half of life.

The descriptive findings from Table 4 thus corroborate the key insight gained from a sequential visualization of career trajectories (Fig. 2): career upward mobility after the first professorship is more characteristic for eminent economists than for eminent sociologists.

### Duration of Professorships

**Fig. 3:**
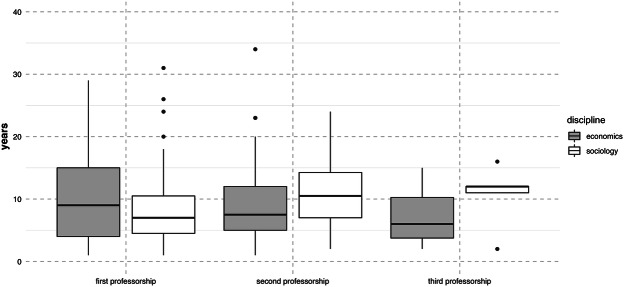
Years spent in a career position. *Notes* The middle of the boxplot indicates the median. The lower and upper hinges correspond to the first and third quartiles (the 25^th^ and 75^th^ percentiles). Outliers, or observations that are located outside the hinges, are represented by points

Differences in career trajectories are not only to be found in the different order of stages, but also in their timing. I hypothesized that if sociologists face more difficulties than economists when aiming to move between different departments, this should manifest itself in longer average durations of different career stages. Figure 3 depicts the median number of years spent in the first three professorships. It becomes visible that economists spend more years in their first professorship, which can be partly explained by the fact that they are appointed professor comparatively early in life (see Table 1). The pattern is reversed regarding the second and third professorship. Economists move more quickly through both career stages.

### Visiting Networks

Two distinct forces shape elite careers: hierarchies and networks. Universities are hierarchical structures in which elites quickly move through lower positions (assistant professor, associate professor) to reach the final career plateau, from which there is nowhere to go professionally within the very same institution. Elites, however, can also entertain multiple affiliations by visiting other research institutions. By doing so, they establish networks between the home institution and other visiting institutions. To obtain a global view of the relatively large visiting networks in both disciplines, I decided to shrink all vertices (i.e., home/visiting institutions) belonging to a certain prestige class to one single vertex. Put more concretely, I shrank, for example, all US departments belonging to the top five ranked departments to a new vertex that is labeled “USA {rank 1–5}”. A visit to a host institution was only counted once per scholar.^18^

**Fig. 4:**
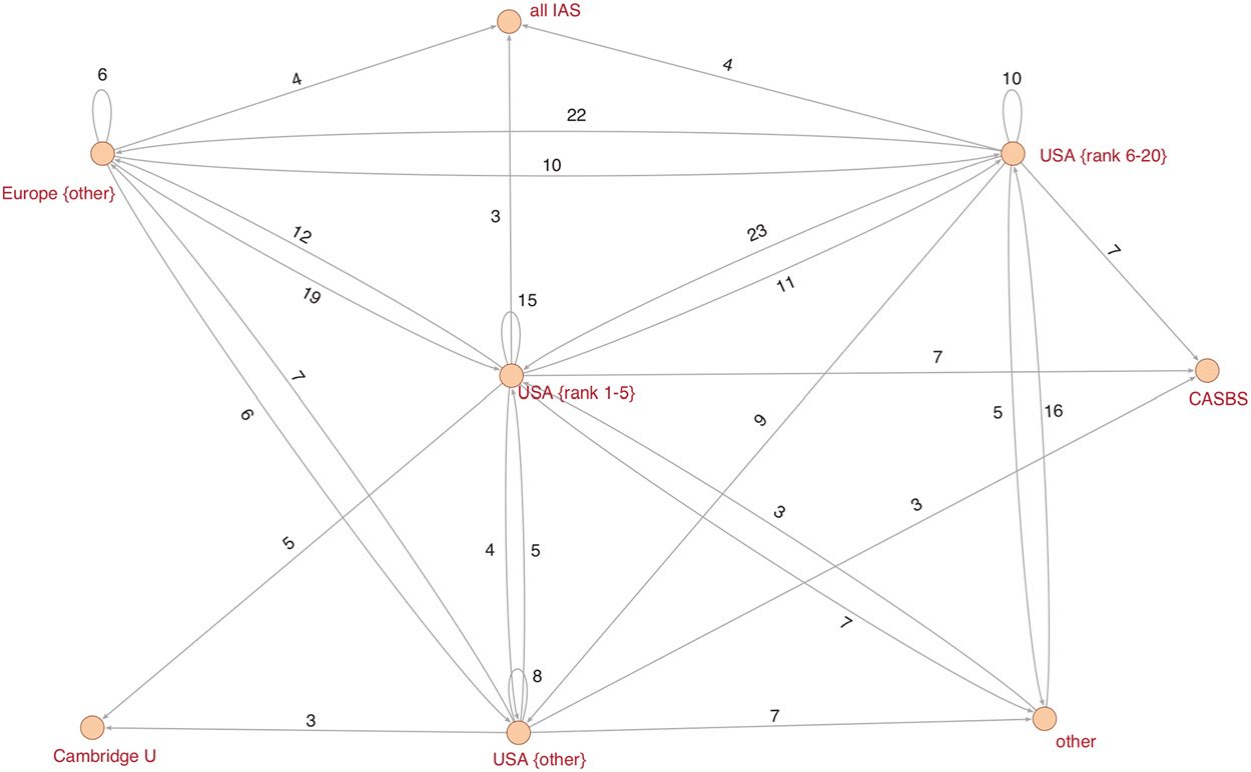
Visiting relationships between research institutions in economics. **Notes**. Arcs indicate the direction of the relationship. Considered are only multiple relationships equal to or greater than 3

In the condensed network depicted in Fig. 4, most scholars leave second-tier US departments (ranks 6–20) to either visit US top-tier departments (ranks 1–5) or conduct studies at European departments. Moreover, there is a significant inflow to the top US departments from Europe as well as a high circulation between departments belonging to the top five. The overall network structure suggests that the ‘big five’ (Chicago, M.I.T., Harvard, Stanford, Yale) are the main magnet in the visiting network, which is confirmed by the indegree distribution that considers individually all departments included in the network (see Appendix A6 in the Online Supplementary Material).

**Fig. 5:**
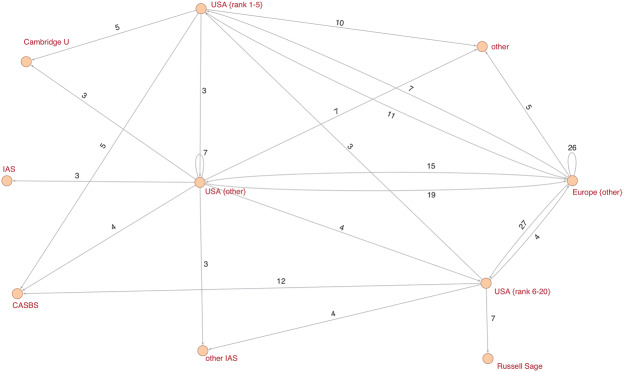
Visiting relationships between research institutions in sociology. **Notes**. Arcs indicate the direction of the relationship. Considered are only multiple relationships equal to or greater than 3

In sociology, the visiting network has European departments as an epicenter (see Fig. 5). A total of 26 scholars circulate between European departments, and 27 scholars temporarily visit second-tier US departments (ranks 6–20). There are also significant inflows from top US departments (Harvard, Berkeley, Chicago) and from rather average US departments. The outdegree distribution of all departments (see Appendix A6 in the Online Supplementary Material) indicates that professors of UC Berkeley hold the most fellowships and visiting professorships. The indegree distribution reveals that research stays at the Center for Advanced Study in the Behavioral Sciences (CASBS) at Stanford University are popular among sociologists as well. The distinctive characteristic of the CASBS is the absence of the usual faculty commitments and the opportunity to focus exclusively on research.

## Conclusions

Rummaging in the graveyard of sociological studies on academic elites, this article has taken up an almost forgotten theoretical claim made by Whitley (1976)—that the elite structure is analogous to the overall structure of a discipline—and subjected it to empirical scrutiny.

Whitley’s theory postulates that discipline-level factors influence the individual careers of academic elites. He highlights what one could call the ‘macro-to-micro effect of discipline-specific degrees of epistemological uncertainty’: variations in the extent to which work procedures, problem definitions, and theoretical goals that are shared between scholars lead to a different organization of disciplines. Disciplines such as economics that are unified under core cognitive goals and methods develop institutionalized specialties and clear hierarchies of reputation (‘umbrella disciplines’). Disciplines where ‘anything goes’ remain fractured with ambiguous reputation hierarchies, specialties that do not become autonomous and institutionalized, and researchers who tend to pursue different goals at different places of knowledge production (‘polytheistic disciplines’). The second part of Whitley’s theory further implies that, given these different cognitive and social configurations, elites will go through a few highly institutionalized career channels in ‘umbrella disciplines,’ while the career pathways of elites in ‘polytheistic disciplines’ remain comparatively undetermined.

Most of the insights gained from a comparative analysis of eminent economists and sociologists are in line with Whitley’s predictions. Economists transition earlier after their Ph.D. to their first professorship. Their careers are, first and foremost, characterized by upward mobility while horizontal career growth is quite common in sociology. Further, economists spend fewer years in subsequent professorships, which might indicate that switching departments is easier where common standards of research evaluation are firmly established. Most importantly, economists clearly aim for professorships and/or research stays at the top five departments of the discipline whereas elite careers in sociology are less bound to the most prestigious research institutions. The only commonality identified between both populations, besides similarities in their age structure and national background, is the nature of PhD-granting departments: Both groups of elites were mainly educated in elite departments.

These results further suggest that elite control is likely to vary between ‘polytheistic’ and ‘umbrella disciplines.’ Up until the 1960s, reputational leaders such as Talcott Parsons and Robert K. Merton were affiliated with few departments (Harvard, Columbia) that dominated the production of PhDs in American sociology and partly, as in the case of Chicago University, managed major publication outlets. However, this concentrated elite power quickly vanished. Elite sociologists such as Jürgen Habermas (Frankfurt University), Immanuel Wallerstein (Binghamton University), Niklas Luhmann (University of Bielefeld) or Manuel Castells (University of Southern California) work(ed) at departments that may offer ideal working conditions, but certainly do not have the largest rosters of highly qualified students or administrative control over critical resources. In contrast, the economics departments at M.I.T., Harvard, Stanford, Princeton, and Chicago that dominate the careers of nearly all Nobel Laureates continue to attract the most talented students and to control the major journals of the discipline (Heckman and Moktan 2018).

Altogether the low-level data reported in this study verify Whitley’s high-level theory: Where there are no unified and universally accepted problem definitions, methodologies, and theoretical approaches, there is also no homogenous academic elite.

### Limitations and Future Research Directions

Identifying eminent sociologists that together present the best possible comparison group was one of the main challenges this author faced, knowing that the Nobel committee invests much effort in searching for deserving economists all over the world. As argued, citations appear in the absence of any *ne plus ultra* award as the most plausible ‘Geiger counter’ for identifying the most eminent sociologists, as it is well established that there is a strong relationship between SSCI citation scores and “virtually every refined measure of quality” (Cole and Cole 1973: 35). However, citations remain only a proxy of peer recognition. Therefore, I tested whether the additional inclusion of eminent sociologists such as Robert N. Bellah, William J. Goode, Arthur Stinchcombe, David A. Snow, Shmuel Eisenstadt, and Claude Lévi-Strauss leads to results substantially different from those reported here. As it turns out, this is not the case.

While this article’s strength is to highlight differences between two populations, its main shortcoming is that it does not explain how exactly distinct elite profiles come about. It was merely suggested that the many competing standards of excellence in sociology make it difficult for selection committees at leading sociology departments (and their advisers) to identify the elite, which in turn introduces randomness into the hiring process of elite departments (Stinchcombe 1994). Economists, on the other hand, can rely on commonly acknowledged indicators of excellence. It is only through interviews with recruiting agents and in-depth studies of the hiring procedures in elite institutions (Caplow and McGee 1961) that the significance of contested and shared epistemological beliefs of academic excellence for elite careers can be examined. If proven important, Whitley’s theory could be extended by incorporating a more detailed account of the forces that make the nexus between the overall intellectual structure of a discipline and the composition of its elite.

## Electronic supplementary material

Below is the link to the electronic supplementary material.

Supplementary material 1 (XLSX 54 kb)

## Footnotes

1 It is exactly this logically interrelatedness of propositions that sets a theory apart from a mere conceptual analysis (Merton 1968).

2 Two examples of scholars who use Whitley´s typology merely to characterize natural sciences are Jagtenberg (1983) and Taylor (1996).

3 In his seminal work *The Intellectual and Social Organization of the Science*, Whitley (1984) does not use the abstract compound word ‘ordering principle’ but rather stresses that there is *uncertainty* over the ‘right’ ways of doing, for example, economics or sociology. He further distinguishes between a ‘technical uncertainty’ (a degree of unpredictability and variability within a field regarding the accepted methods to solve empirical problems) and a ‘strategic uncertainty’ (consensus or lack of consensus on which problems are important and, thus, on how to gain reputation).

4 In another contribution, Whitley differentiates between two variants of institutionalization with regards to specialities. Specialties become cognitively institutionalized if there is high consensus on the definition of phenomena and on appropriate analytical techniques, and socially institutionalized if researchers communicate mostly within specialties only (Whitley 1974).

5 Whitley (1984: 106) stresses this difference in elite control explicitly: “Contemporary Anglo-Saxon sociology and economics, for instance, differ greatly in the influence a relatively small number of intellectual leaders is able to exert over research strategies and significance criteria.”

6 The main reason for my narrow focus on professorships is that detailed information on assistant and associate professorships is often not available. Elites often do not indicate in their official CVs when exactly they switched from an untenured position (assistant professor) to a tenured position (associate position). However, the methodology applied in this article, sequence analysis, builds on rather specific temporal information.

7 When it comes to the question of what had been regarded as a prize-worthy contribution, “it is probably correct to say that the selection committee has looked, in particular, at the originality of the contribution, its scientific and practical importance, and its impact on scientific work” (Lindbeck 2001: 212). In hindsight, it also becomes apparent that the prize has been awarded for one or several specific contributions (such as the Prize for R. C. Merton or M. Friedman) as well as for lifetime contributions (such as the Prize for P. Samuelson or S. Kuznets).

8 That high citation scores indicate elite status becomes evident in the case of Nobel Laureates in economics: “The Swedish Academy tended to award the prize close to the winners´ citation peak, i.e. at the very height of the winners´ reputations” (Bjork et al. 2014: 193).

9 The main justification for a three-dimensional measure of eminence is that *some* scholars elicit extremely high citation scores not necessarily because of the quality of their work but rather because of the bandwagon status of a single (methodological) publication, or because they are working in fields in which publications are in general more often cited than in other areas.

10 See www.webofknowledge.com.

11 Karl Marx, Thorstein Veblen, Georg Simmel, George H. Mead, and Max Weber are excluded from the analysis to achieve a similar age distribution between the population of eminent economists and sociologists.

12 An issue that necessitates reflection is that the authors’ CVs might seek to maximize credibility (Dietz et al. 2000). According to the author´s experience, however, top scholars mostly view the CV as a (historical) record for chronicling outputs and activities.

13 The archive hosts its biographical material online: http://agso.uni-graz.at/lexikon/.

14 See for an outline of the methodology: https://www.topuniversities.com/qs-world-university-rankings/methodology.

15 In many cases, however, it is impossible to know the exact length of stay. The curriculum vitae of Christopher A. Sims, for example, reveals that he was a visiting professor at Yale University in 1974. We can only assume that such information implies that Sims spent at least one semester at Yale (and not less time).

16 Only in the case of F. Hayek does it appear impossible to unambiguously identify the country of residence. In Table 2 Hayek therefore falls into the mixed category ‘United Kingdom/USA.’

17 For results that consider the age structure in each discipline, please contact the author.

18 The sociologist Jeffrey Alexander, for example, visited the Swedish Collegium for Advanced Study in the Social Sciences twice during his time at UCLA, namely, in 1992 and in 1996. In our analysis, however, this results only in a simple link between UC-Los Angeles and the Swedish Collegium.
